# Gram-Negative Infections in Adult Intensive Care Units of Latin America and the Caribbean

**DOI:** 10.1155/2014/480463

**Published:** 2014-11-27

**Authors:** Carlos M. Luna, Eduardo Rodriguez-Noriega, Luis Bavestrello, Manuel Guzmán-Blanco

**Affiliations:** ^1^Pulmonary Division, Department of Medicine, José de San Martin Hospital, University of Buenos Aires, Arenales 2557, Piso 1, Dep. A, 1425 Buenos Aires, Argentina; ^2^Hospital Civil de Guadalajara “Fray Antonio Alcalde” and Institute of Infectious and Experimental Pathology, University Center of Health Sciences, University of Guadalajara, Guadalajara, JAL, Mexico; ^3^Clinica Reñaca, Viña Del Mar, Chile; ^4^Private Hospital Medical Center of Caracas and Vargas Hospital of Caracas, Caracas, Venezuela

## Abstract

This review summarizes recent epidemiology of Gram-negative infections in selected countries from Latin American and Caribbean adult intensive care units (ICUs). A systematic search of the biomedical literature (PubMed) was performed to identify articles published over the last decade. Where appropriate, data also were collected from the reference list of published articles, health departments of specific countries, and registries. Independent cohort data from all countries (Argentina, Brazil, Chile, Colombia, Cuba, Mexico, Trinidad and Tobago, and Venezuela) signified a high rate of ICU infections (prevalence: Argentina, 24%; Brazil, 57%). Gram-negative pathogens, predominantly *Acinetobacter baumannii, Klebsiella pneumoniae, Pseudomonas aeruginosa*, and *Escherichia coli*, accounted for >50% of ICU infections, which were often complicated by the presence of multidrug-resistant strains and clonal outbreaks. Empirical use of antimicrobial agents was identified as a strong risk factor for resistance development and excessive mortality. Infection control strategies utilizing hygiene measures and antimicrobial stewardship programs reduced the rate of device-associated infections. To mitigate the poor health outcomes associated with infections by multidrug-resistant Gram-negative bacteria, urgent focus must be placed on infection control strategies and local surveillance programs.

## 1. Introduction

Recognition that critically ill patients receive greater medical attention and have better health outcomes when they are grouped together in one patient care center gave rise to the intensive care unit (ICU) in 1952 [1]. An unintended consequence of housing critically ill patients together in ICUs is increased risk for infection [2–5], the epidemiology of which has been studied more extensively in North America, Europe, and Australasia [2–10] than in Latin America [11].

Between 3% and 12% of hospitalized patients in developed countries acquire a health care-associated infection (HAI) [12]. Of all HAIs, at least one-quarter occur in ICUs [13, 14]. In the extended prevalence of infection in intensive care (EPIC II) study, infection was independently associated with an increased risk of hospital death; the ICU mortality rate of infected patients was more than twice that of noninfected patients (25% versus 11%, resp.; *P* < 0.001), as was the hospital mortality rate (33% versus 15%, resp.; *P* < 0.001) [5].

Gram-negative bacteria represent the most common nosocomial isolates, primarily* Pseudomonas aeruginosa*,* Escherichia coli*,* Klebsiella* spp., and* Acinetobacter* spp. [5]. Infection by Gram-negative pathogens is complicated by emergence and global spread of strains expressing numerous mechanisms for antimicrobial resistance. The probability of encountering such a pathogen is far higher in the ICU than in other patient care areas [15].

This review describes the epidemiology of Gram-negative infections in Latin American and Caribbean adult ICUs by country over the last 10 years.

## 2. Materials and Methods

A systematic search of the biomedical literature was conducted. MEDLINE (via PubMed) was searched, limited by the dates of June 6, 2002, to March 9, 2014, for articles using the following terms and Boolean logic: (“intensive care unit” OR “ICU” OR “hospital” OR “nosocomial”) AND (“Gram-negative infection” OR “Gram-negative pathogen” OR “bacilli”) AND (“Latin America” OR “South America” OR “Central America” OR “Mexico” OR “Guatemala” OR “Honduras” OR “Nicaragua” OR “Costa Rica” OR “El Salvador” OR “Belize” OR “Panama” OR “Colombia” OR “Venezuela” OR “Guyana” OR “Suriname” OR “French Guiana” OR “Brazil” OR “Ecuador” OR “Peru” OR “Bolivia” OR “Paraguay” OR “Uruguay” OR “Chile” OR “Argentina”). In addition, the same search strategy was conducted this time featuring 27 countries and overseas territories of the Caribbean. No delimiters were applied to the search strategy. The subject matter of all citations yielded from the search was screened by the authors for relevance. We were particularly interested in observational studies (retrospective and prospective) that reported information on the frequency, morbidity, and mortality of ICU infections by Gram-negative bacteria, the phenotypic and genotypic characteristics of these bacteria, and risk factors for acquiring such infections. Except for studies reporting on clonality, only those reporting information on >50 patients were summarized. In addition, we used data from http://www.provenra.com.ve/ which publishes clinical microbiologic findings specific to Venezuela.

## 3. Results

### 3.1. Literature Search Results

Twenty-five observational studies were identified and selected for review. The features and properties of these studies are summarized in the Supplementary Table (see Supplementary Material available online at http://dx.doi.org/10.1155/2014/480463). Most studies pertained to ICUs in Brazil (*n* = 11), followed by Argentina (*n* = 5), Colombia (*n* = 3), Chile (*n* = 2), Cuba (*n* = 1), Mexico (*n* = 1), Trinidad and Tobago (*n* = 1), and Venezuela (*n* = 1).

### 3.2. ICU Infection Frequency

Table 1 shows that a range of different study designs were utilized to estimate the epidemiology of ICU infections in Argentina and Brazil. The prevalence of HAIs in adult ICUs was 24% in a pooled analysis of 2 multicenter, observational, cross-sectional studies conducted within the framework of Argentina's National Surveillance of Hospital Infections Program in 2004 and 2005 [16]. The most frequently occurring infection was pneumonia (43%), followed by primary bloodstream infection (21%) and urinary tract infection (13%). The incidence of ventilator-associated pneumonia (VAP) microbiologically confirmed by significant growth (>10^4^ colony forming units/mL in bronchoalveolar lavage culture) was 15% in a study performed in ICUs of 6 hospitals in the metropolitan area of Buenos Aires during 1999 to 2001 [17].

In Brazil, a study conducted in 19 ICUs at the Hospital das Clínicas, University of São Paulo, São Paulo, and which had a similar design to EPIC II [5] reported an overall infection rate of 57% [18]. This finding is comparable with the 60% point prevalence estimated in EPIC II for Central/South America [5]. Thirty-one percent of the Hospital das Clínicas infections were acquired in the ICU, yielding an ICU HAI point prevalence of 17% [18]. The ICU HAI rate was slightly lower in a recent (2005–2008) prospective observational study conducted in Minas Gerais (14%) [23]. In prospective observational studies, the prevalence of VAP in Brazil ranged from 27% to 66% [19–22].

### 3.3. Frequency of Gram-Negative Bacterial Infections

**Figure 1:**
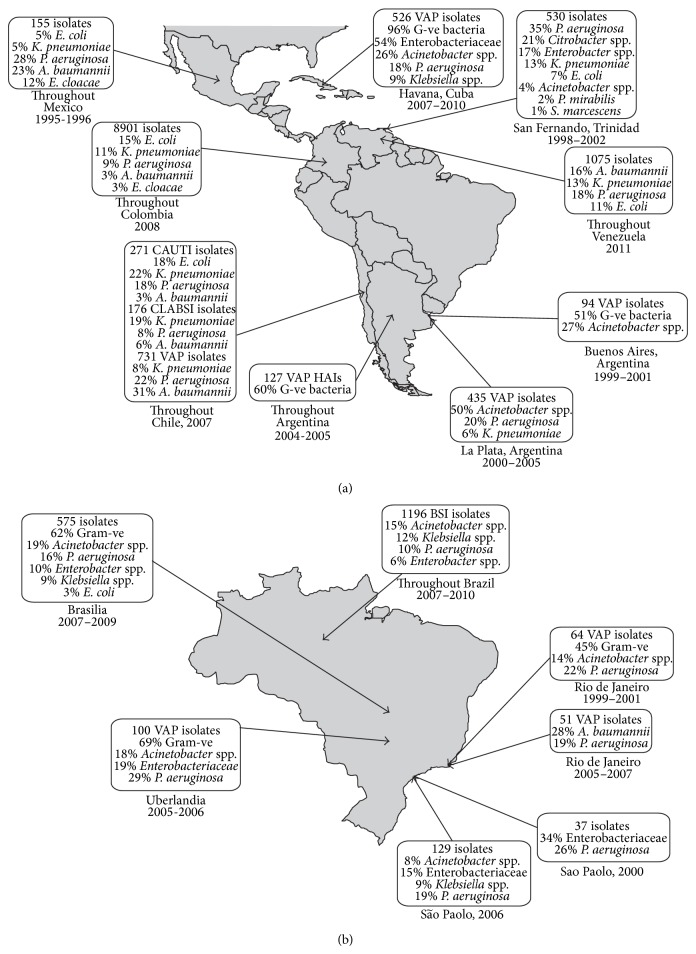
Frequency of Gram-negative bacteria (all* n*/*N*) among bacteriologically documented infections in (a) Latin American and Caribbean ICUs and (b) Brazilian ICUs specifically [16–22, 28, 33–36, 42, 66]. VAP: ventilator-associated pneumonia, CAUTI: catheter-associated urinary tract infection, CLABSI: central line-associated bloodstream infection, HAI: hospital-acquired infection, BSI: bloodstream infection, and ICU: intensive care unit.

Figure 1 shows that Gram-negative bacteria were the predominant infectious agents in Latin American and Caribbean ICUs, although polymicrobial infections were not unusual [16, 17, 22, 33–36].* Acinetobacter* spp.,* Klebsiella* spp.,* and P. aeruginosa* were the 3 most frequently encountered pathogens in isolates collected throughout the continent, and* Acinetobacter* spp. particularly so in VAP isolates [16]. Time-trend data pertaining to 741 mechanically ventilated patients with suspected nosocomial pneumonia were available between 2007 and 2010 for the 3 ICUs of the Hermanos Ameijeiras Hospital, Havana, Cuba [35]. The most prevalent bacterial infections were* Acinetobacter* spp. (26%),* Pseudomonas* spp. (18%), and* Klebsiella* spp. (9%), which represented a 1% decrease in the* Pseudomonas* spp. prevalence rate and a 4% and 11% increase in prevalence of* Acinetobacter* spp. and* Klebsiella* spp., respectively [35]. The distribution of infectious agents in the ICU of San Fernando, Trinidad, was different to that in ICUs of other countries in that* Citrobacter* spp. (21%) and* Enterobacter* spp. (17%) represented the second and third most frequently isolated pathogens after* P. aeruginosa* (35%). When these data were stratified by anatomical collection site,* P. aeruginosa*,* K. pneumoniae*,* Citrobacter* spp., and* Enterobacter* spp. were the predominant isolates from sputum, while from urine it was* P. aeruginosa* and* K. pneumoniae* and from blood it was* Citrobacter* spp.

### 3.4. Susceptibility Data

**Figure 2:**
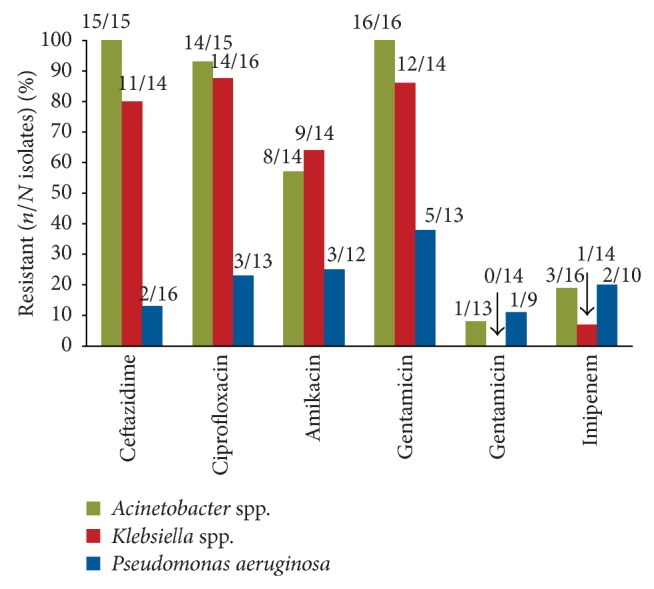
Resistant Gram-negative bacilli. Resistance profile of Gram-negative bacilli isolated from adult intensive care patients who participated in Argentina's National Surveillance of Hospital Infections Program in 2004 and 2005 [16].

Variable but clinically significantly high rates of multidrug resistance were observed throughout Latin America. Of the small number of isolates collected by Argentina's National Surveillance of Hospital Infections Program in 2004 and 2005 that underwent phenotypic profiling, most strains of* Acinetobacter* spp. and* Klebsiella* spp. were resistant to ceftazidime, ciprofloxacin, amikacin, and gentamicin (Figure 2). Furthermore, their susceptibility to the carbapenems, meropenem and imipenem, was compromised [16]. Retrospective data collected from the ICUs of 3 hospitals in Buenos Aires demonstrated that of 61 episodes of VAP caused by* Acinetobacter* spp. or* P. aeruginosa* only 30 isolates were carbapenem susceptible and 31 were colistin-only susceptible [25].

In Brazil, findings from a 10-year prospective study conducted between 1999 and 2008 in a 716-bed (including 89 beds in ICUs) tertiary university hospital (São José do Rio Preto, SP, Brazil) showed that 6314 of 9416 multidrug-resistant Gram-negative bacteria isolated from hospitalized patients were from patients in the ICU (*P* < 0.001) [37]. In rank order, the 3 most common multidrug-resistant Gram-negative bacteria were* A. baumannii*,* P. aeruginosa*, and* K. pneumoniae*. A major finding from the study was the marked increase in prevalence of multidrug-resistant Gram-negative bacteria from study commencement (332 isolates in 1999) to termination (1221 isolates in 2008). The assertion probably holds true for the ICU given the disproportionate number of multidrug-resistant Gram-negative bacteria detected in this patient care area [37].

More detailed antimicrobial susceptibility data comes from the Brazilian Surveillance and Control of Pathogens of Epidemiological Importance (SCOPE) study, which collected 2447 isolates from patients with bloodstream infections (half of whom were in an ICU) [33]. Cephalosporins, aminoglycosides, fluoroquinolones, and carbapenems were not active against >50% of* Acinetobacter* spp. isolates tested, in which the *β*-lactamase OXA-23 (*bla*_OXA-23_) gene was detected in 85 of 112 (76%) isolates tested. More than one-third of the* P. aeruginosa* isolates were resistant to commonly used antimicrobial agents, including imipenem and meropenem. The *bla*_IMP_ gene was detected in 6 (10%) isolates and the *bla*_SPM_ gene was detected in 24 (41%) isolates out of 59 carbapenem-resistant* P. aeruginosa* isolates. Similarly, high proportions of* Klebsiella* spp. were resistant to ampicillin/sulbactam, piperacillin/tazobactam, ceftazidime, and cefepime (54%, 34%, 54%, and 50%, resp.). Resistance to imipenem and meropenem was observed in 0.3% and 1.3% of the isolates, respectively. Of the 94 extended spectrum *β*-lactamase- (ESBL-) positive isolates tested, *bla*_TEM_, *bla*_CTX_, and *bla*_SHV_ genes were present in 84 (89%), 86 (91%), and 68 (72%) strains, respectively. Carbapenem resistance was associated with harboring of the *bla*_KPC_ gene (*K. pneumoniae* carbapenemase (KPC)) [33].

Resistance rates among the 14 cultures positive for* P. aeruginosa* at the Hospital das Clínicas, São Paulo, Brazil, were 50% for both ceftazidime and gentamicin, 42% for both ciprofloxacin and amikacin, and 30% for imipenem [18]. Similarly, in 2 separate ICUs in Rio de Janeiro hospitals, multidrug-resistant organisms were identified in 43% of culture-positive patients with VAP in 1 ICU [19], while half of all* A. baumannii* strains collected from patients with VAP were resistant to carbapenems in the other [22].

**Figure 3:**
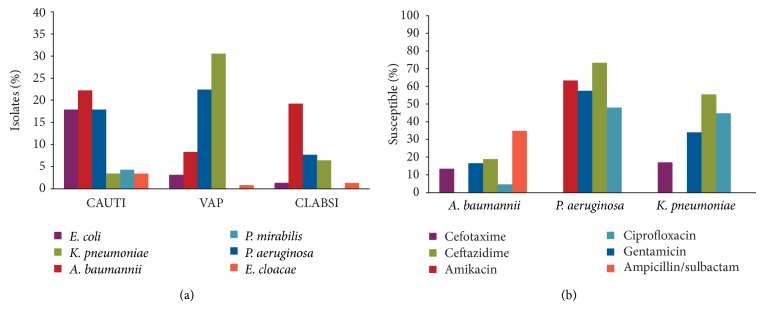
Gram-negative organisms (Chilean ICUs). The proportion of susceptible* A. baumannii* (*n* = 159),* P. aeruginosa* (*n* = 173), and* K. pneumoniae* (*n* = 135) isolates collected from all 31 hospitals [34]. CAUTI: catheter-associated urinary tract infection, VAP: ventilator-associated pneumonia, CLABSI: central line-associated bloodstream infection, and ICU: intensive care unit.

In Chile, low levels of antimicrobial susceptibility were found for* A. baumannii* and* Klebsiella pneumoniae* isolates while* P. aeruginosa* susceptibility ranged from 48% (for ciprofloxacin) to 73% (for amikacin; Figure 3) [34]. In a separate Chilean study conducted in 2007, susceptibility data describing 454* A. baumannii* ICU isolates collected by an independent network showed that the percentage of isolates susceptible to imipenem and meropenem was 62% and 57%, respectively; this was far lower than in other patient care areas (83% and 84%, resp.). Similarly, lower levels of* P. aeruginosa* susceptibility (*N* = 716 isolates) to both antimicrobials occurred in ICUs (54% and 58%, resp.) than in other patient-care areas (78% and 77%, resp.) [38].

In Colombia, temporal WHONET 5.4 (World Health Organization Collaborating Centre for Surveillance of Antimicrobial Resistance, Boston, MA, USA) antimicrobial resistance data for Gram-negative bacilli isolated from 14 ICUs from 2006 until 2008 belonging to the Colombian Nosocomial Resistance Study Group was available [39]. Antimicrobial resistance frequencies of* K. pneumoniae* and* E. cloacae* to third-generation cephalosporins remained steady over the 3-year study period (range, 20–41%), while resistance of* E. coli* to third-generation cephalosporins showed a decreasing trend (ceftazidime, 6% to 2%; ceftriaxone, 8% to 7%; both *P* ≤ 0.001). Rodríguez et al. [40] reported simultaneous increased percentages of* K. pneumoniae* resistance toimipenem (1.3% to 4.0%), ciprofloxacin (10% to 14%), and cefotaxime (28% to 31%) during 2007 to 2009; increases also were noted in ceftazidime-resistant strains of* E. coli* (8% to 10%) and imipenem-resistant strains of* A. baumannii* (56% to 63%), with reduced percentages of ciprofloxacin-resistant* E. coli* (28% to 26%), ceftazidime-resistant* P. aeruginosa *(31% to 24%), and ciprofloxacin-resistant* P. aeruginosa* (28% to 24%; *P* < 0.01).

Of 155 bacterial strains isolated from 119 nosocomial infections in Mexico, 89 were nonfermenting Gram-negative rods resistant to all commonly used drug classes except ticarcillin/clavulanic acid (45%) and imipenem (16%) [24]. The 43 Enterobacteriaceae were slightly more susceptible, with 52% of isolates resistant to cefotaxime, 21% to cefpirome, 47% to aztreonam, 40% to amikacin, 19% to ciprofloxacin, and 2% to imipenem [24].

**Figure 4:**
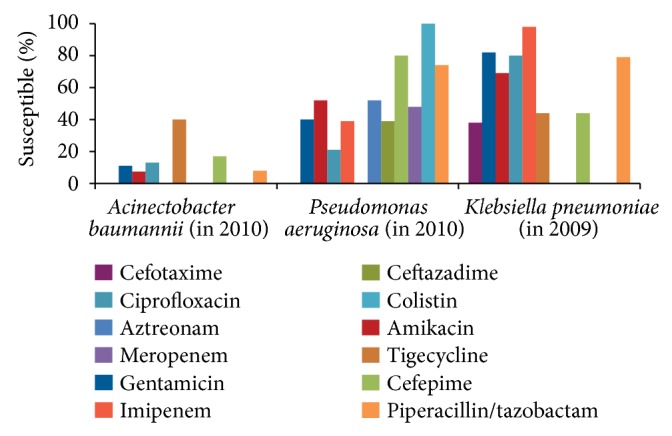
Gram-negative organisms (Venezuelan intensive care units). Susceptibility of Gram-negative bacilli isolated from adult intensive care patients who participated in the Venezuelan Surveillance Program on Antimicrobial Resistance [16].

In Venezuela, blood cultures of patients in the ICU during 2011 indicated that* P. aeruginosa* (18%),* A. baumannii* (16%),* K. pneumoniae* (13%), and* E. coli* (11%) were the most common Gram-negative organisms isolated (Figure 1) [36]. High levels of resistance of* P. aeruginosa* and* K. pneumoniae* to some antimicrobialshave been documented (Figure 4) [36]. All isolates of* A. baumannii* remained susceptible to colistin while most* P. aeruginosa* isolates were susceptible to cefepime (80%). Resistance to the aminoglycosides, amikacin and gentamicin, and ciprofloxacin was high among* A. baumannii* and* P. aeruginosa *[36].

Susceptibility data were available for two Caribbean countries [41, 42]. In Cuba,* Acinetobacter* spp. showed high levels of resistance to commonly used antimicrobial drug classes; 2% of isolates were resistant to colistin.* Pseudomonas* spp. and* Klebsiella* spp. were highly resistant to ampicillin/sulbactam and had moderate or low resistance to other agents [41]. Data from the same hospital revealed that tigecycline and colistin were the only antibiotics fully effective against* A. baumannii* strains isolated in 2011 patients with VAP; only colistin was fully effective against* P. aeruginosa* strains [41]. In Trinidad by contrast, isolates were relatively antimicrobial susceptible although these data are now >10 years old [42]. Of the 10 antimicrobial agents tested, the highest rate of resistance was for ampicillin (88%) and the lowest rates were for imipenem (6%), ciprofloxacin (6%), and piperacillin-tazobactam (12%). The most common isolate* P. aeruginosa* was susceptible to >82% of the antimicrobials [42].

### 3.5. Mortality and Risk Factors

Inappropriate and tardy antimicrobial therapies have been associated with resistance and, to a lesser extent, higher mortality in patients in Latin American ICUs [17, 24–28, 43].  Table 2 also shows that a previous ICU stay and a previous episode of VAP predispose to infections by multidrug-resistant Gram-negative bacteria [25–28].

In Argentina, the VAP mortality rate due to any pathogen was higher among 46 patients who had received prior antibiotics than among 17 patients who had not received antibiotics (59% versus 29%, *P* = 0.075) [17]. Prior antimicrobial treatment in this study was defined as current use of antimicrobials or previous use of antimicrobials for >24 hours during the 10 days before the diagnosis of VAP [17]. Findings of a later study by the same investigators showed that the overall mortality rate in VAP was 29% in the 24 patients who received therapy that provided coverage against all isolated pathogens at VAP onset (i.e., appropriate therapy) compared with a rate of 64% in the 52 patients who received either inadequate therapy or delayed initiation of appropriate therapy (*P* = 0.007) [43]. In another Argentinian study, prior antimicrobial therapy for >10 days and a previous episode of VAP remained significantly associated with colistin-only susceptible VAP; 41% of VAP by colistin-only susceptible strains, but none of those by carbapenem-susceptible strains had received prior carbapenem therapy [25].

In Mexico, a multivariate analysis of prospective, nested, case-control data collected from 4 ICUs in Mexico from 1995 to 1996 indicated that inadequate antibiotic treatment and development of VAP were major risk factors for the 59% crude mortality rate (Table 2) [24]. The term inadequate antibiotic treatment was not limited to initial empirical antibiotic therapy but included failure to administer proper antibiotics according to in vitro susceptibility results [24]. Although antibiotic resistance in Gram-negative rods was not an independent risk factor for mortality in this study, there was a strong association between antibiotic resistance and inadequate treatment [24].

The phenotype of* A. baumannii* was a risk factor for death on day 30 of hospitalization in a Colombian study, in which HAIs by multidrug-resistant strains were associated with significantly greater 30-day mortality than drug-sensitive strains (42% versus 9%, resp.; *P* = 0.0074) [44]. This difference was maintained when the patients' risk factors were evaluated by multivariate analysis.

Mortality associated with Gram-negative infection approximated 33% in San Fernando, Trinidad, with all fatalities occurring in patients with pneumonia and bloodstream infections [42]. No formal analysis was conducted in this study to detect any association between mortality and antimicrobial resistance rates; however, resistance to the most commonly used antimicrobials was high and correlated with consumption [42].

### 3.6. Clonal Outbreaks

A review of the molecular epidemiology of clones in the Latin American hospital setting has been reviewed elsewhere [45, 46]. Our literature findings indicated that outbreaks of infections within ICUs are often owing to a small number of clones (Table 3).* A. baumannii* pulsed-field gel electrophoresis clone I has been widespread in several Buenos Aires hospitals since 1981, and carbapenem-resistant pulsed-field gel electrophoresis clone IV became prevalent in the 1990s [47]. Multidrug-resistant epidemic and endemic clones of* Acinetobacter* spp. and* P. aeruginosa* emerged in Brazilian ICUs during the late 1990s [48, 49]. In the Meropenem Yearly Susceptibility Test Information Collection (MYSTIC) program conducted in 5 ICUs in São Paulo and Brasilia during 2002, 36 multidrug-resistant* P. aeruginosa* isolates were clustered into 5 genotypes [29]. Two carbapenem-resistant* P. aeruginosa* outbreaks were reported in the ICUs of Brazilian teaching hospitals; 1 in 2001 (Hospital Universitário São Francisco, São Paulo, Brazil) and the other between 2003 and 2005 (Minas Gerais, Brazil) [30, 31]. As detected in the general hospital setting [45, 46], a nosocomial outbreak of KPC-producing* K. pneumoniae* featured a Buenos Aires ICU with a high capacity for dissemination and a high mortality rate [32].

## 4. Discussion

Based on the published information garnered, which was heterogeneous with respect to study collection dates and methodology, the overall prevalence rate of infections in Brazilian ICUs ranged from 31% to 66%, with pneumonia/VAP prevalence tending to be the most frequent type of infection. Data from Argentinian and Brazilian ICUs indicated that the prevalence of infections acquired in ICUs was 17% to 24%. Infections in ICUs throughout the Latin American and Caribbean countries evaluated reflect a preponderance of Gram-negative pathogens and the dissemination of multidrug-resistant strains, including carbapenem-resistant strains of* Acinetobacter* spp.,* P. aeruginosa*, and* K. pneumoniae*. Genes encoding ESBLs, KPC, and metallo-*β*-lactamase among some of these organisms can be considered a major public health problem. Risk factors for Gram-negative infections were similar to those found in other countries and included previous or inappropriate empiric antimicrobial therapy. In three ICUs, inadequate antibiotic treatment of Gram-negative infections was a risk factor for mortality. It should be noted, however, that the only information available on inadequate antimicrobial treatment is related to previous therapy, current empirical therapy, or a current change to empirical therapy. No information was available regarding the effect of deescalating antimicrobial therapy on resistance and mortality, which would have better characterized the associations. Shortening the duration of antimicrobial therapy may be possible for those VAP patients exhibiting a good outcome based on serial measurements of clinical pulmonary infection score [17], and deescalation is now recognized as one component of an optimal care strategy in patients with sepsis [50].

Mortality stemming from device-associated Gram-negative infections in Latin American and Caribbean ICUs underscores the need for the development, implementation, and reinforcement of infection control strategies. We recommend use of a sequential checklist to remind health care personnel of simple measures to reduce the opportunity for nosocomial infection. Use of such a checklist was associated with a sustained reduction in central line-associated bloodstream infections over an initial 18-month period [51], and the benefits persisted at 36 months after implementation [52]. Similar checklists in the form of the so-called “prevention bundles” in the United States have aided the prevention of ICU VAP [52, 53] and led to the development of a unique checklist for the prevention of surgical infections. The World Health Organization has adopted this safe surgery checklist and has recommended its application worldwide [54, 55].

Limitations of results include lack of data available for most countries in Latin America, as well as bias in data reported from different centers or networks. For instance, most data were obtained from a few tertiary centers in each country, which likely report elevated resistance rate relative to their national averages. Methodology of data reporting, collection, and analysis also may differ among laboratories, countries, and surveillance networks.

The high rates and continent-wide dissemination of multidrug-resistant Gram-negative bacteria means that empiric prescribing of antimicrobials (without cultures) cannot be recommended. Furthermore, findings from our literature review showed a clear and direct association between antimicrobial use and resistance development in Argentina, Brazil, Cuba, Mexico, and Trinidad. Thus, diagnostic and treatment decisions should be made based on the local susceptibility patterns within each institution, framed within regional, national, and global surveillance programs. Phenotypic methods are useful in identifying bacterial isolates at the genus and species level and provide the clinician with an antimicrobial resistance profile to guide therapy [56]. More discriminating phenotypic techniques such as 3-dimensional testing are required, given the exponential increase in Gram-negative bacteria harboring ESBLs, KPCs, and metallo-*β*-lactamase. It is important to note that clinical microbiologic laboratories routinely fail to identify pathogens harboring multiple *β*-lactamases, inducible AmpC-encoded *β*-lactamases, and KPCs [57–59].

In practice, critically ill patients receiving appropriate antimicrobial therapy (i.e., empirical therapy modified due to clinical response or pathogen identification) have better health outcomes than those receiving inappropriate therapy (i.e., unmodified empirical treatment) [60]. Similarly, Luna et al. [61] studied 283 patients that were mechanically ventilated for ≥48 hours to determine if biweekly routine endotracheal aspirate (ETA) cultures were more effective for managing VAP than the American Thoracic Society/Infectious Diseases Society of America guidelines [62]. Unless the sample was available ≤2 days of the onset of VAP, the guidelines-based approach was more accurate than the ETA-based approach for prescribing initial empiric antibiotics. Fewer days of antimicrobial therapy resulted when ETA cultures were considered.

Finally, we recommend use of antimicrobial stewardship programs with instructions on limiting inappropriate antimicrobial use, administering the correct dose via the correct route of administration, and the optimum duration of therapy [63]. We also encourage antimicrobial recycling and restricting certain antimicrobial agents for specific indications [64]. When properly implemented, the programs result in significant changes in the prevalence of bacterial resistance with attendant reductions in morbidity, mortality, and costs [63–65]. Despite the financial and logistic difficulties associated with implementing the components of antimicrobial stewardship programs, we recommend the pursuit of guidelines for antimicrobial use and formulary restrictions with appropriate review and feedback clauses in Latin America and the Caribbean [64].

## 5. Conclusion

There are high infection rates by Gram-negative bacteria in Latin American and Caribbean ICUs aggravated by spread of multidrug-resistant bacterial strains and clonal outbreaks. High rates of morbidity and mortality will prevail unless modifiable risk factors are better controlled.

## Supplementary Material

The features and properties of the 25 observational studies conducted in Latin American and Caribbean ICUs that were identified and selected for our review are included in a supplementary table. Most studies pertained to ICUs in Brazil (n = 11), followed by Argentina (n = 5), Colombia (n = 3), Chile (n = 2), Cuba (n = 1), Mexico (n = 1), Trinidad and Tobago (n = 1), and Venezuela (n = 1).

## Figures and Tables

**Table 1:** Infection rates in ICUs of Argentinian and Brazilian hospitals.

Reference	Study design	Location	Year of study	Number of patients	Rate of infection
Lossa et al. 2008 [16]	Cross-sectional, multicenter	Argentina nationwide	2004 and 2005	356	(i) Pooled prevalence, 24% (95% CI, 20%–29%) (a) Pneumonia, 43% (b) BSI, 21% (c) UTI, 13%

Luna et al. 2003 [17]	Prospective, multicenter	Buenos Aires, Argentina	1999–2001	472	(i) VAP incidence, 63/472 (13%)

Toufen Junior et al. 2003 [18]	1-day point prevalence	São Paulo, Brazil	2000	126	(i) Overall prevalence, 72/126 (57%) (a) CAI, 15/72 (21%) (b) Non-ICU nosocomial infection, 24/72 (33%) (c) ICU-acquired infection, 22/72 (31%) (d) Undefined, 11/72 (15%)

de Queiroz Guimarães and Rocco 2006 [19]	Prospective observational	Rio de Janeiro, Brazil	1999–2001	278	(i) VAP prevalence, 38% (36 cases/1000 ventilator days)

Lima et al. 2007 [20]	Prospective observational	São Paulo, Brazil	2006	71	(i) Prevalence, 47/71 (66%)

da Rocha et al. 2008 [21]	Prospective observational	Uberlândia, Brazil	2005-2006	275	(i) VAP prevalence, 31% (25 cases/1000 ventilator days)

Rodrigues et al. 2009 [22]	Prospective observational	Rio de Janeiro, Brazil	2005–2007	233	(i) VAP prevalence, 27% (17 cases/1000 ventilator days)

de Oliveira et al. 2010 [23]	Prospective observational	Minas Gerais, Brazil	2005–2008	2300	(i) CAI, 437/2300 (19%) (a) 284 (12%) patients colonized by resistant microorganisms during ICU hospitalization, 61% of whom developed an infection (ii) Nosocomial infection, 311/2300 (14%) (a) 84/311 (27%) owing to resistant pathogens

ICU: intensive care unit, CI: confidence interval, BSI: bloodstream infection, UTI: urinary tract infection, VAP: ventilator-acquired pneumonia, and CAI: community-acquired infection.

**Table 2:** Multivariate analysis of risk factors for crude mortality and acquisition of drug-resistant Gram-negative infections in Latin American ICUs.

Outcome	Risk factor	OR (95% CI)	*P* value	Reference
Crude mortality	Inadequate antibiotic treatment	70.5	<0.00001	Zaidi et al. 2002 [24]
	Development of VAP	7.7	0.004	

Colistin-susceptible VAP	Overall ICU stay, 40 days	31.6 (31.5–495.9)	0.014	
	Duration of prior antimicrobial therapy >10 days	13.2 (2.2–78.7)	0.005	Rios et al. 2007 [25]
	Previous episode of VAP	6.0 (1.0–35.7)	0.047	

Imipenem-resistant *P. aeruginosa *	Previous ICU stay	3.54 (1.3–9.7)	0.03	Furtado et al. 2009 [26]

HAP by imipenem-resistant *P. aeruginosa *	Piperacillin/tazobactam	14.31 (1.0–200.2)	0.04	Furtado et al. 2010 [27]
	Third-generation cephalosporin	7.45 (1.8–30.9)	0.006	

Antimicrobial resistance to *Acinetobacter spp.* and *P. aeruginosa *	Prior exposure to antimicrobial agents	NR	<0.05	Weyland et al. 2011 [28]

ICU: intensive care unit, OR: odds ratio, CI: confidence interval, VAP: ventilator-associated pneumonia, HAP: hospital-acquired pneumonia, and NR: not reported.

**Table 3:** Molecular epidemiology of Gram-negative clones in intensive care units of Latin America.

Reference	Location, year	Clonal type	Antimicrobial susceptibility	Mechanism of antimicrobial resistance	Notes
Figueiredo-Mendes et al. 2005 [29]	São Paulo, Brazil (4 centers) and Brasília, Brazil (1 center), 2002	36 *P. aeruginosa* isolates (clones A, B, C, D, and G)	Multidrug resistant; carbapenem MIC, ≥32 *µ*g/mL	MBL production (except for clone D1 and D2)	Interhospital spread probably owing to transfers of infected patients, share of health care workers, and exchange of medical equipment among institutions

Gales et al. 2004 [30]	Hospital Universitário São Francisco, São Paulo, Brazil, 2001	5 carbapenem-resistant* P. aeruginosa *strains from 4 patients (clone B and C_1_)	Clone B susceptible to all classes except carbapenems; clone C_1_ susceptible to polymyxin B only	NR	Dissemination may have been owing to cross-contamination. Clone C_1_ had a similar PGFE pattern to clone C_2_ (previously isolated from Hospital São Paulo, São Paulo, Brazil)

Cezário et al. 2009 [31]	University Hospital in Minas Gerais, Brazil, 2003–2005	36 multidrug *P. aeruginosa* isolates (clones A, B, C, and D)	Multidrug resistant	MBL-producing strains were positive for *bla*_SPM-1_	Strong temporal/spatial relationship indicated cross-contamination

Córdova et al. 2012 [32]	Hospital Dr. Cosme Argerich, Buenos Aires, Argentina, 2009-2010	6 patients infected with KPC-producing *K. pneumoniae *ST258	Susceptible to tigecycline and colistin only	KPC	Attributable mortality, 26%; ST258 had a high capacity for dissemination

MIC: minimum inhibitory concentration, MBL: metallo-*β*-lactamase, NR: not reported, PGFE: pulsed gel field electrophoresis, *bla*_SPM-1_: *β*-lactamase SPM-1 gene, and KPC: *K. pneumoniae* carbapenemase.
