# Supplementary material for: Gram-Negative Infections in Adult Intensive Care Units of Latin America and the Caribbean
Source: Crit Care Res Pract. 2014 Nov 27;2014:480463. doi: 10.1155/2014/480463 (PMC4265515; doi:10.1155/2014/480463)
Supplement: Supplementary file 1 — The features and properties of the 25 observational studies conducted in Latin American and Caribbean ICUs that were identified and selected for our review are included in a supplementary table. Most studies pertained to ICUs in Brazil (n = 11), followed by Argentina (n = 5), Colombia (n = 3), Chile (n = 2), Cuba (n = 1), Mexico (n = 1), Trinidad and Tobago (n = 1), and Venezuela (n = 1). [file 480463.f1.pdf]

Supplementary Table. Standard features of the observational studies conducted in Latin American and Caribbean ICUs, which were identified from the literature search and selected by the authors according to predefined criteria (i.e., *n* > 50 patients or isolates; dates, June 6, 2002–June 20, 2013).

Reference	Study design	Location	No. of ICUs	Collection period	Anatomical collection site	No. and type of patients (or isolates)
ARGENTINA						
Luna et al. 2003 [17]	Prospective, multicenter	Buenos Aires	6	1999–2001	Respiratory tract	472 MV patients hospitalized for > 72 hours
Luna et al. 2006 [36]	Prospective, multicenter	Buenos Aires	6	1999–2003	Respiratory tract	76 MV patients with bacteriologically confirmed VAP
Barbola et al. 2008 [43]	10-week, prospective cohort, single center	Buenos Aires	1	July–Sept 2003	Air, environment, and staff hands	1042 screening samples
Rios et al. 2007 [28]	Retrospective, multicenter	Buenos Aires	3	2001–2004	Respiratory tract	61 episodes of VAP caused by *Acinetobacter spp* or *P. aeruginosa* (30 by carbapenem-susceptible and 31 by colistin-susceptible isolates)
Lossa et al. 2008 [16]	Cross-sectional, multicenter	Nationwide	26 in 2004; 27 in 2005	May–June 2004; May–June 2005	Blood, respiratory tract, urine	158 admissions in 2004; 201 admissions in 2005
Weyland et al. 2011 [39]	Prospective, case-control	Buenos Aires	1	2000–2005	Respiratory tract	435 isolates from 430 patients
BRAZIL						
Toufen Jr. et al. 2003 [18]	1-day point prevalence,	São Paulo	19	2000	Blood, respiratory	126 occupying a bed in a 24-hour period

	multicenter				tract, urine, surgical wounds	
Martins de Queiroz Guimarães et al. 2006 [21]	Prospective, single-center	Rio de Janeiro	1	1999–2001	Respiratory tract	278 on MV for > 24 hours
Furtado et al. 2009 [38]	Retrospective, case-control, single-center	São Paulo	1	2003–2004	Any site	63 imipenem-resistant *P. aeruginosa* cases > 48 hours after admission versus 182 controls
Lima et al. 2007 [22]	Prospective, single-center	São Paulo	1	2006	Blood, respiratory tract, urine	71 occupying a bed in a 24-hour period and followed until discharge or death
de Andrade da Rocha et al. 2012 [20]	Prospective, single-center	Uberlândia	1	2005–2006	Respiratory tract	275 on MV for > 48 hours
Rodrigues et al. 2009 [23]	Prospective, single-center	Rio de Janeiro	1	2005–2007	Respiratory tract	233 on MV for > 48 hours
Rubio et al. 2013 [29]	Prospective, single-center	São José do Rio Preto	NR (89 beds)	1999–2008	All sites	10,732 multidrug resistant Gram-negative bacterial isolates collected hospital wide
de Oliveira et al. 2010 [19]	Prospective, single-center	Minas Gerais	1	2005–2008	Blood, respiratory tract, urine, surgical wounds	2300 admissions with a stay of > 24 hours
Furtado et al. 2009 [37]	Retrospective, case-control, single-center	São Paulo	1	2006–2008	All sites	58 imipenem-resistant *P. aeruginosa* cases > 48 hours after admission versus 47 imipenem-susceptible *P. aeruginosa* cases

						versus 182 controls
Marra et al. 2011 [24]	Prospective, multicenter	Nationwide	~16	2007–2010	Blood	1257 with ≥ 1 culture of blood sampled ≥ 48 hours after admission yielding a pathogenic organism
Cartaxo Salgado et al. 2011 [66]	Retrospective, case control, single-center	Brasilia	1	2007–2009	NR	401 patients
CHILE						
Bustamante et al. 2007 [25]	Prospective, multicenter	Nationwide	39 with urine data; 38 with MV data; 43 with BSI data	2007	Blood, respiratory tract, urine	10,237 with a permanent urinary catheter; 5265 on MV; 13,411 with a CVC
Silva et al. 2011 [30]	Prospective, multicenter, independent network	Nationwide	14	2009	All sites	≥ 984 isolates
COLOMBIA						
Briceño et al. 2010 [31]	Prospective, multicenter	Nationwide	14	2006–2008	All sites	22,344 isolates
Villalobos Rodríguez et al. 2011 [32]	Retrospective, multicenter	Nationwide	79	2007–2009	All sites	25,915 isolates
Lemos et al. 2011 [40]	Prospective, multicenter	Bogotá	3	2006–2010	All sites	62 with HAI multidrug-resistant *A. baumannii* and 103 patients with drug-sensitive *A. baumannii* infection ≥ 48 hours after

						admission
CUBA						
Medell et al. 2012 [26]	Prospective, single-center	Havana	3	2007–2010	Respiratory tract	741 on MV
MEXICO						
Zaidi et al. 2002 [33]	Prospective, nested case control, multicenter	Yucatán, Mexico City, and Michoacán	4	1995–1996	All sites	113 who acquired a HAI (67 cases who died and 46 controls who survived)
TRINIDAD AND TOBAGO						
Orrett, 2004 [35]	Prospective, single-center	San Fernando, Trinidad	1	1998–2002	All sites but primarily sputum, urine, wound and blood (93%)	530 consecutive surgical and medical patients

ICU: intensive care unit, MV: mechanical ventilation, VAP: ventilator-associated pneumonia, NR: not reported, BSI: bloodstream infection, CVC: central venous catheter, HAI: health care–associated infection.
